# Overexpressing the *cpr1953* Orphan Histidine Kinase Gene in the Absence of *cpr1954* Orphan Histidine Kinase Gene Expression, or Vice Versa, Is Sufficient to Obtain Significant Sporulation and Strong Production of *Clostridium perfringens* Enterotoxin or Spo0A by *Clostridium perfringens* Type F Strain SM101

**DOI:** 10.3390/toxins16040195

**Published:** 2024-04-18

**Authors:** Iman Mehdizadeh Gohari, Jessica L. Gonzales, Francisco A. Uzal, Bruce A. McClane

**Affiliations:** 1 Department of Microbiology and Molecular Genetics, University of Pittsburgh School of Medicine, Pittsburgh, PA 15219, USA; imehdiza@pitt.edu; 2 California Animal Health and Food Safety Laboratory System, School of Veterinary Medicine, University of California Davis, San Bernardino, CA 92408, USA; jlygonzales@ucdavis.edu (J.L.G.); fauzal@ucdavis.edu (F.A.U.)

**Keywords:** *Clostridium perfringens*, Spo0A, sporulation, orphan histidine kinase, enterotoxin

## Abstract

The CPR1953 and CPR1954 orphan histidine kinases profoundly affect sporulation initiation and *Clostridium perfringens* enterotoxin (CPE) production by *C. perfringens* type F strain SM101, whether cultured in vitro (modified Duncan–Strong sporulation medium (MDS)) or ex vivo (mouse small intestinal contents (MIC)). To help distinguish whether CPR1953 and CPR1954 act independently or in a stepwise manner to initiate sporulation and CPE production, *cpr1953* and *cpr1954* null mutants of SM101 were transformed with plasmids carrying the *cpr1954* or *cpr1953* genes, respectively, causing overexpression of *cpr1954* in the absence of *cpr1953* expression and vice versa. RT-PCR confirmed that, compared to SM101, the *cpr1953* mutant transformed with a plasmid encoding *cpr1954* expressed *cpr1954* at higher levels while the *cpr1954* mutant transformed with a plasmid encoding *cpr1953* expressed higher levels of *cpr1953*. Both overexpressing strains showed near wild-type levels of sporulation, CPE toxin production, and Spo0A production in MDS or MIC. These findings suggest that CPR1953 and CPR1954 do not function together in a step-wise manner, e.g., as a novel phosphorelay. Instead, it appears that, at natural expression levels, the independent kinase activities of both CPR1953 and CPR1954 are necessary for obtaining sufficient Spo0A production and phosphorylation to initiate sporulation and CPE production.

## 1. Introduction

*Clostridium perfringens* is a Gram-positive, rapidly-growing, spore-forming, rod-shaped, anaerobic, fermentative bacterium of the phylum Firmicutes [1,2,3]. This bacterium is widely-dispersed in nature, including such environments as sewage, decaying vegetation, and soil [1,4,5,6]. Although *C. perfringens* is an important human and animal pathogen [7], it can also be found in normal human and animal intestinal microbiota [1,4].

The virulence of *C. perfringens* involves its ability to produce potent exotoxins. This bacterium secretes a multitude (more than 20) of different toxins, which allows it to cause a broad array of diseases. Those diseases occur in both humans and other animals and range from myonecrosis (gas gangrene) to enterocolitis and fatal enterotoxemia (involving damage to non-intestinal organs caused by toxins absorbed into the circulation from the intestines) [7,8].

*C. perfringens* strains are currently categorized into seven types (A–G) based upon their carriage of genes encoding six toxins [2]. By definition, *C. perfringens* type F isolates must harbor the alpha toxin and enterotoxin (*cpe*) genes but cannot carry the genes encoding the other four typing toxins [2]. Type F isolates cause human food-poisoning and human non-foodborne gastrointestinal illnesses such as antibiotic-associated diarrhea and sporadic diarrhea [1,9,10,11,12,13,14]. Epidemiological studies, as well as molecular Koch’s postulate analyses, have supported the importance of enterotoxin (CPE) production for the virulence of type F *C. perfringens* isolates [1,15]. 

Besides producing toxins, another feature of *C. perfringens* is its ability to form spores that are metabolically inert and highly resistant to harsh environmental conditions [16]. In addition, production of CPE is strictly dependent upon sporulation [16,17]. Therefore, sporulation plays a critical role in the survival, transmission, and pathogenicity of type F isolates. 

In *C. perfringens*, like other Clostridia and *Bacillus* spp., a hierarchical cascade of regulation is required for sporulation and, subsequently, *cpe* expression driven by sporulation-associated sigma factors [17,18,19,20,21]. Sporulation initiation is mediated by the master transcriptional regulator Spo0A, which is activated by phosphorylation [22]. In *Bacillus* spp., Spo0A is activated through a phosphorylation process involving a multicomponent phosphorelay [23,24]. However, that phosphorelay is absent from *C. perfringens* [25,26,27], leading to proposals that *C. perfringens* initiates sporulation by phosphorylating Spo0A using sporulation-specific orphan histidine kinases (OHKs), i.e., histidine kinases encoded by genes not located with genes encoding a cognate response regulator. To evaluate that hypothesis, nucleotide and protein BLAST analyses were performed [26,28] to identify putative OHKs in SM101, which is a transformable derivative of *C. perfringens* type F food poisoning strain NCTC 8798 [17]. Those searches indicated that the SM101 genome encodes seven apparent OHKs, which are designated CPR0195, CPR1055, CPR1316, CPR1493, CPR1728, CPR1953, and CPR1954. Interestingly, while these putative OHKs are unique to *C. perfringens*, they are encoded by almost all genome-sequenced *C. perfringens* isolates available in the GenBank database [26,28]. 

In 2019, it was reported [26] that disrupting the *cpr0195* gene caused a partial, but significant, decrease in sporulation and CPE production by SM101 when grown in modified Duncan–Strong laboratory sporulation medium (MDS). However, MDS cultures of an isogenic *cpr1055* null mutant still sporulated and produced CPE at similar levels as wild-type SM101, demonstrating that not all putative orphan histidine kinases are required for sporulation and CPE production in MDS cultures. This study also showed that the purified kinase domain of CPR0195 can directly phosphorylate Spo0A in vitro [26]. 

More recently [28], the remaining five putative orphan kinase genes (*cpr1316*, *cpr1493*, *cpr1728*, *cpr1953*, and *cpr1954*) were disrupted in SM101 and those null mutants, along with the *cpr0195* and *cpr1055* null mutants prepared by Freedman et al. [26], were used to investigate the contribution, if any, of each apparent OHK to sporulation and CPE production under two different culture conditions, i.e., MDS or mouse intestinal contents (MIC) as a pathophysiologically relevant ex vivo model. The results identified several phenotypic patterns. The CPR1055 and CPR1728 OHKs are not required for sporulation or CPE production whether SM101 is grown in MDS or MIC. In contrast, CPR0195, CPR1316, and CPR1493 are important for sporulation and CPE production when SM101 is grown in MDS but they are unnecessary for either sporulation or CPE production when SM101 is cultured in MIC. Finally, CPR1953 and CPR1954 are virtually essential for sporulation and CPE production by SM101, whether cultured in MDS or MIC [28]. 

Our previous work [28] revealed that the *cpr0195*, *cpr1316* and *cpr1493* null mutants make less Spo0A protein compared to wild-type SM101. Furthermore, the *cpr1953* and *cpr1954* null mutants of SM101 produced much less Spo0A than even those other OHK null mutants. While CPR0195 was shown to directly phosphorylate Spo0A in vitro [26], this ability has not yet been examined for the CPR1316, CPR1493, CPR1953, or CPR1954 OHKs. However, studies using Phos-Tag gels demonstrated that the *cpr1954* null mutant cannot activate the reduced amount of Spo0A that it does produce [28]; whether CPR1954 OHK directly or indirectly mediates Spo0A phosphorylation remains undetermined. Collectively, the decreased levels of Spo0A production by the *cpr1953* and *cpr1954* null mutants and lack of Spo0A phosphorylation (at least for the *cpr1954* null mutant) offer explanations for the defective sporulation and CPE production of these OHK mutants. 

Importantly, it remains unclear how CPR1953 and CPR1954 function to initiate sporulation and CPE production, i.e., do they have redundant, but additive, functions or must they function together stepwise, for example as a novel phosphorelay? To help distinguish between those possibilities, the current study evaluated the effects on SM101 sporulation and CPE production when the *cpr1953* gene is overexpressed in the absence of *cpr1954* expression or the *cpr1954* gene is overexpressed in the absence of *cpr1953* expression. 

## 2. Results

### 2.1. Construction and Characterization of cpr1953 or cpr1954 Overexpressing Strains 

For this study, we prepared an SM101 derivative overexpressing *cpr1954* in a background free of *cpr1953* expression and a second SM101 derivative overexpressing *cpr1953* in a background free of *cpr1954* expression. This was accomplished by transforming the previously constructed [28] CPR1953KO and CPR1954KO strains (where “KO” represents isogenic null mutant) with plasmids carrying, respectively, either the *cpr1954* or *cpr1953* genes. Specifically, CPR1953KO was transformed by electroporation with the previously prepared [28] plasmid pJIR750-*cpr1954COM* (carrying the *cpr1954* gene), which restored near wild-type levels of sporulation and CPE production when transformed into CPR1953KO. Similarly, CPR1954KO was transformed with pJIR750-*cpr1953COM* (carrying the *cpr1953* gene), which restored near wild-type levels of sporulation and CPE production when transformed into CPR1954KO. The resultant strains were named CPR1953KO-1954COM and CPR1954KO-1953COM.

Our previous study [28] had also prepared two plasmids named pJIR750-*cpr1954COM*-H410A and pJIR750-*cpr1953COM*-H430A, where the codon encoding a critical His residue in the predicted phosphotransfer region of, respectively, CPR1954 or CPR1953 was replaced by a codon encoding an alanine substitution. Those substitutions rendered the variant proteins unable to restore sporulation or CPE production when pJIR750-*cpr1954COM*-H410A or pJIR750-*cpr1953COM*-H430A, respectively, were transformed into the CPR1954KO or CPR1953KO mutants [28]. Therefore, as a control to evaluate whether the kinase activity of CPR1953 or CPR1954 is important for the phenotypes of CPR1953KO-1954COM and CPR1954KO-1953COM, the CPR1953KO and CPR1954KO mutants were also transformed, respectively, with pJIR750-*cpr1954COM*-H410A or pJIR750-*cpr1953COM*-H430A to create CPR1953KO-1954COM-H410A and CPR1954KO-1953COM-H430A.

**Figure 1:**
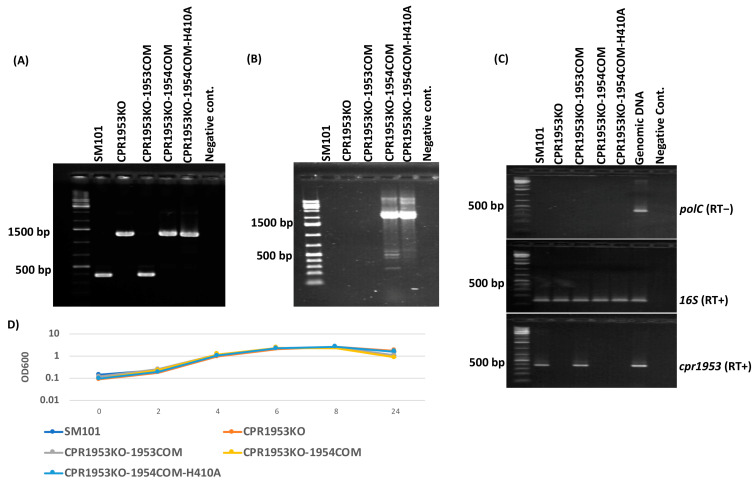
Preparation and characterization of CPR1953KO-1954COM and CPR1953KO-1954COM-H410A. (**A**) PCR assay verifying that CPR1953KO-1954COM and CPR1953KO-1954COM-H410A carry intron disruptions in their *cpr1953* gene. PCR was conducted using internal *cpr1953* gene primer sets which amplified a larger amplicon from CPR1053KO (1265 bp) versus SM101 wild-type strain (365 bp) due to insertion of a 900 bp group II intron into the specific DNA target site of the *cpr1953* gene. The complementing strain CPR1953KO-1953COM amplified a PCR product similar in size to that of wild-type SM101. As expected, the overexpressing transformants amplified amplicons similar in size to that of isogenic CPR1953KO using the same primer sets, confirming those strains still carried a disrupted *cpr1953* gene. (**B**) PCR assay confirmed the presence of pJIR750-based plasmids in transformants using specific primers that were able to amplify PCR products from contiguous pJIR750 and *cpr1954* sequences. As shown, no PCR products were observed for SM101, CPR1953KO, and CPR1953KO-1953COM. (**C**) RT-PCR analyses for *polC* gene (**top**), *16S* gene (**middle**), or *cpr1953* gene (**bottom**) transcription by wild-type SM101, CPR1953KO, CPR1953KO-CPR1953COM, CPR1953KO-1954COM, and CPR1953KO-1954COM-H410A strains. Purified genomic DNA was used as a positive control. (**D**) Growth curve analysis for wild-type SM101 and its derivative strains cultured in MDS medium at 37 °C for different time points. Error bars depict SDs. All data were collected from three independent repetition. *p* value < 0.05 was considered as significant differences among the results.

**Figure 2:**
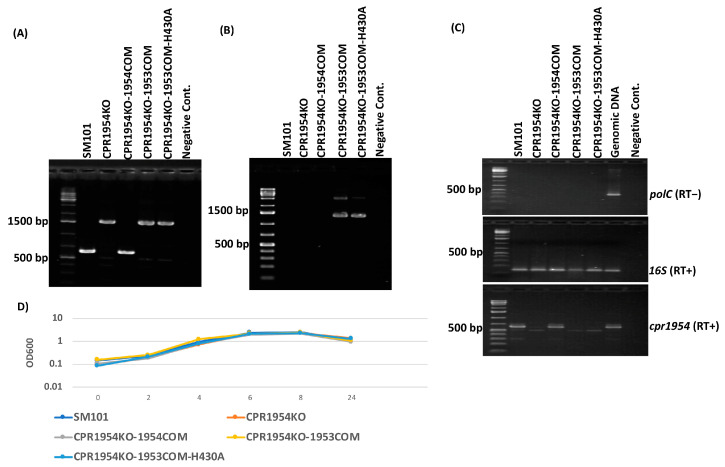
Preparation and characterization of CPR1954KO-1953COM and CPR1954KO-1953COM-H430A strains. (**A**) PCR assay verifying CPR1954KO-1953COM and CPR1954KO-1953COM-H430A carry intron disruptions in their *cpr1954* genes. PCR was conducted using internal *cpr1954* gene primer sets which amplified a larger amplicon from CPR1954KO (1496 bp) versus wild-type SM101 (596 pb) due to insertion of a 900 bp group II intron into the specific DNA target site of the *cpr1954* gene. The complementing strain CPR1954KO-1954COM amplified a PCR product similar to that of the wild-type SM101 strain. As expected, the CPR1954KO-1953COM and CPR1954KO-1953COM-H430A transformants amplified products similar in size to that of isogenic CPR1954KO using the same primer sets, confirming those strains still carry intron disruptions in their *cpr1954* gene. (**B**) PCR assay confirmed the presence of pJIR750-based plasmids in these transformants using specific primers, which were able to amplify PCR products from contiguous pJIR750 and *cpr1953* sequences. As shown, no PCR products were observed for SM101, CPR1954KO, and CPR1954KO-1954COM. (**C**) RT-PCR analyses for transcription of the *polC* gene (**top**), *16S* gene (**middle**), or *cpr1954* gene (**bottom**) by wild-type SM101, CPR1954KO, CPR1954KO-CPR1954COM, CPR1954KO-1953COM, and CPR1954KO-1953COM-H430A. Purified genomic DNA was used as a positive control. (**D**) Growth curve analysis for wild-type SM101 and its derivative strains cultured in MDS medium at 37 °C for different time points. Error bars depict SDs. All data were collected from three independent repetitions. *p* value < 0.05 was considered as significant differences among the results.

To verify that the newly constructed strains still carried disruptions in their relevant OHK gene, PCR assays were conducted using primers specific for internal sequences upstream and downstream of the intron insertion site in the *cpr1953* or *cpr1954* open reading frame (ORF). Using DNA from wild-type SM101 (Figure 1A and Figure 2A), the *cpr1953* primer pair amplified a 365 bp amplicon, while the *cpr1954* primer pair amplified a 596 bp amplicon. The same internal primers amplified a larger amplicon using DNA from strains carrying the *cpr1953* or *cpr1954* null mutations due to the targeted insertion of a 900 bp intron into, respectively, their *cpr1953* or *cpr1954* genes (Figure 1A and Figure 2A). Specifically, PCR using primers to internal *cpr1953* ORF sequences amplified an ~1265 bp product using DNA from CPR1953KO, CPR1953KO-1954COM, or CPR1953KO-1954COM-H410A (Figure 1A). Similarly, PCR using primers to internal *cpr1954* ORF sequences amplified a 1496 bp product using DNA from CPR1954KO, CPR1954KO-1953COM, or CPR1954KO-1953COM-H430A (Figure 2A). For CPR1953KO-1953COM and CPR1954KO-1954COM, PCR using the same primer sets to internal ORF sequences in *cpr1953* or *cpr1954*, respectively, amplified a product matching the size of the PCR product using DNA from wild-type SM101, confirming transformation (Figure 1A and Figure 2A). 

To confirm that the newly constructed transformants carry, as specified, the pJIR750-*cpr1953COM*, pJIR750-*cpr1954COM*, pJIR750-*cpr1954COM*-H410A, or pJIR750-*cpr1953COM*-H430A plasmids, PCR assays were performed using specific primer sets that included a pJIR750 forward primer and a reverse primer to either *cpr1953* or *cpr1954* ORF sequences. As shown in Figure 1B and Figure 2B, PCR products were amplified for these newly prepared transformant strains, but not SM101 or the *cpr1953* or *cpr1954* null mutants, verifying creation of the correct constructs.

Reverse Transcription-PCR (RT-PCR) was performed to substantiate the loss of expression of each mutated kinase gene. Initially, the quality and purity of each extracted RNA sample was confirmed by performing RT-PCR for expression of the *16S* and *polC* genes, using a PCR reaction performed in the presence or absence of reverse transcriptase (Figure 1C and Figure 2C). Using the same RNA preparations, RT-PCR then demonstrated *cpr1953* expression by SM101 and CPR1953KO-1953COM but not by CPR1953KO, CPR1953KO-1954COM, or CPR1953KO-1954COM-H410A (Figure 1C). Similarly, *cpr1954* expression was detected in SM101 and CPR1954KO-1954COM but not by CPR1954KO, CPR1954KO-1953COM, or CPR1954KO-1953COM-H430A (Figure 2C). 

Finally, the growth phenotype of each *C. perfringens* strain in MDS sporulation medium was evaluated at different time points. Those analyses indicated that the growth rate of all mutants, as well as their derivative transformants carrying pJIR750-based plasmids, was similar to that of wild-type SM101 (Figure 1D and Figure 2D). 

### 2.2. Evidence for Overexpression of the cpr1953 or cpr1954 Genes in, Respectively, CPR1954KO-1953COM or CPR1953KO-1954COM

Expression levels of the *cpr1953* or *cpr1954* genes were then compared between CPR1954KO-1953COM or CPR1953KO-1954COM vs. wild-type SM101 to assess whether these were overexpressing strains. Since qRT-PCR primers could not be designed for *cpr1953* or *cpr1954*, RT-PCR was performed using purified RNA from wild-type SM101 and its derivative OHK null mutants or transformants carrying pJIR750-based plasmids. 

**Figure 3:**
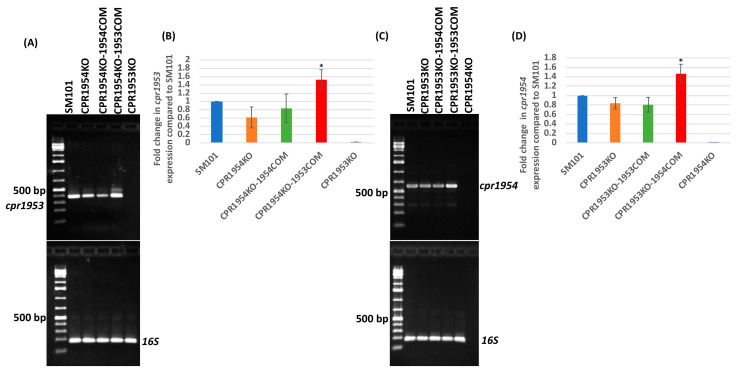
Comparing the expression levels of the *cpr1953* and *cpr1954* genes in various strains. (**A**) RT-PCR comparing the expression levels of the *cpr1953 gene* between SM101, CPR1954KO, CPR1954KO-1954COM, CPR1954KO-1953COM, and CPR1953KO. (**B**) Comparison of *cpr1953* expression levels in SM101 versus its derivative isogenic null mutants or complementing strains as determined by densitometry using Image J software (version 1.53t). (**C**) RT-PCR comparing the expression levels of *cpr1954* between SM101, CPR1953KO, CPR1953KO-1953COM, CPR1953KO-1954COM, and CPR1954KO. (**D**) Comparison of *cpr1954* expression levels in SM101 versus its derivative strains as determined by densitometry using Image J software. In this assay, RT-PCR for 16S RNA was used as loading control. Figure is shown as a representation of three independent experiments. * *p* < 0.05 compared to SM101.

Results of those RT-PCR analyses demonstrated that CPR1953KO-1954COM and CPR1954KO-1953COM expressed their *cpr1954* or *cpr1953* genes, respectively, at significantly higher levels compared to SM101 (Figure 3A–D). No significant difference was noted in *cpr1953* expression levels between SM101 vs. CPR1954KO or CPR1954KO-1954COM (Figure 3A,B). Similarly, no significant differences were apparent in *cpr1954* expression levels between SM101 vs. CPR1953KO or CPR1953-1953COM (Figure 3C,D). In this experiment, all strains were shown by RT-PCR to express similar levels of the *16S* RNA gene, used as a loading control (Figure 3A,C). 

### 2.3. Overexpression of cpr1953 or cpr1954 Genes, Respectively, by the cpr1954 or cpr1953 Null Mutants Is Sufficient to Restore Sporulation and CPE Production to near Wild-Type Levels in MDS Cultures

**Figure 4:**
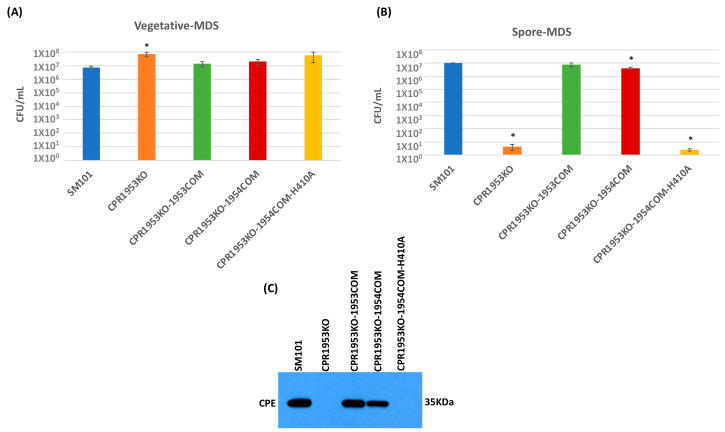
Evaluating the effect of overexpressing the *cpr1953* gene in the absence of *cpr1954* expression on vegetative cell viability, spore formation, and CPE production when SM101 and its derivative strains were cultured in MDS. (**A**) Viable vegetative cell numbers (CFU/mL) when SM101, CPR1953KO, CPR1953KO-1953COM, CPR1953KO-1954COM, or CPR1953KO-1954COM-H1410A were anaerobically cultured overnight at 37 °C in MDS. (**B**) Heat-resistant spore counts (CFU/mL) in aliquots of those same MDS cultures after heating at 70 °C for 20 min to kill the remaining vegetative cells and induce spore germination. (**C**) Western blot analysis of CPE levels in supernatants of those same overnight MDS cultures for SM101, CPR1953KO, CPR1953KO-1953COM, CPR1953KO-1954COM, and CPR1953KO-1954COM-H1410A. The size of the protein band is shown at right. Error bars depict SDs. All data were collected from three independent repetitions. Asterisk represents *p* < 0.05 versus SM10. Western blot is representative of three independent experiments.

**Figure 5:**
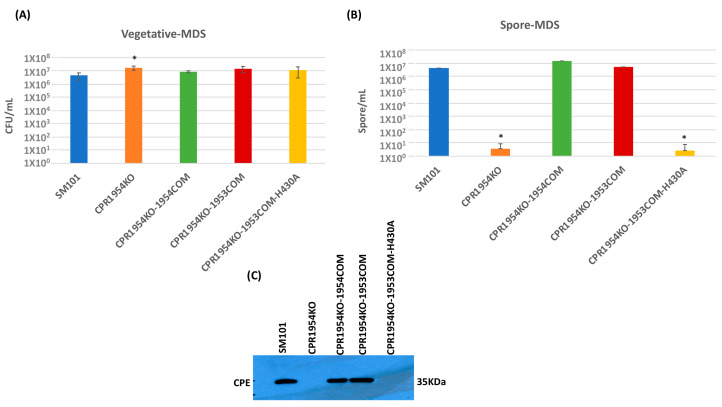
Evaluating the effect of overexpressing the *cpr1954* gene in the absence of *cpr1953* expression on vegetative cell viability, spore formation, and CPE production when SM101 and its derivative strains were cultured in MDS. (**A**) Viable vegetative cell numbers (CFU/mL) when SM101, CPR1954KO, CPR1954KO-1954COM, CPR1954KO-1953COM, and CPR1954KO-1953COM-H430A were anaerobically cultured overnight at 37 °C in MDS medium. (**B**) Heat-resistant spore counts (CFU/mL) in aliquots of those same MDS cultures after heating at 70 °C for 20 min to kill the remaining vegetative cells and spore germination induction. (**C**) Western blot analysis of CPE levels in supernatants of those overnight MDS cultures for SM101, CPR1954KO, CPR1954KO-1954COM, CPR1954KO-1953COM, and CPR1954KO-1953COM-H430A. The size of the protein band is shown at right. Error bars depict SDs. All data were collected from three independent repetition. Asterisk represents *p* < 0.05 versus SM101. Western blot is representative of three independent experiments.

An experiment then evaluated whether overexpression of *cpr1953* in the absence of *cpr1954* expression, or overexpression of *cpr1954* in the absence of *cpr1953* expression, is sufficient to affect vegetative cell viability, spore formation, or CPE production when SM101 is cultured in MDS. Consistent with previous findings [28], overnight MDS cultures of CPR1953KO and CPR1954KO produced slightly, but significantly, more viable vegetative cells compared to wild-type SM101 (Figure 4A and Figure 5A). Overnight MDS cultures of CPR1953KO-1954COM, CPR1953KO-1954COM-H410, CPR1954KO-1953COM, and CPR1954KO-1953COM-H430 did not contain significantly more viable vegetative cells compared to SM101 MDS cultures (Figure 4A and Figure 5A). 

In those overnight MDS cultures, the CPR1953KO mutant produced virtually no spores (Figure 4B), as expected from our previous study [28]. As we also reported previously, complementation of the CPR1953KO mutant to restore *cpr1953* expression significantly enhanced sporulation back to the same levels as detected for SM101 MDS cultures. Importantly, these experiments also revealed for the first time that overexpressing *cpr1954* in the absence of *cpr1953* expression was sufficient to obtain significant sporulation, i.e., MDS cultures of CPR1953KO-1954COM produced 10^6^-fold more spores than CPR1953KO, although the sporulation efficiency of this overexpressing strain was slightly lower than that of wild-type SM101 cultured in MDS (Figure 4B). Demonstrating the involvement of CPR1954 kinase activity for CPR1953KO-1954COM-induced sporulation, MDS cultures of CPR1953KO-1954COM-H410 still produced very low levels of sporulation, similar to CPR1953KO (Figure 4B). 

In MDS, CPE production by those strains closely corresponded to their sporulation phenotype. Unlike the strong CPE production exhibited by SM101 MDS cultures, MDS cultures of CPR1953KO made no detectable CPE, while MDS cultures of the CPR1953KO-1953COM complementing strain made CPE at levels similar to wild-type SM101 (Figure 4C). The CPR1953KO-1954COM strain overexpressing *cpr1954* in the absence of *cpr1953* also made significantly more CPE than CPR1953KO when cultured in MDS, although CPR1953KO-1954COM produced slightly less CPE compared to wild-type SM101 when both are cultured in MDS (Figure 4C). Under the same MDS growth condition, the CPR1953KO-1954COM-H410 strain produced no detectable CPE (Figure 4C). 

Similarly, when cultured in MDS, CPR1954KO sporulated extremely poorly (<10 spores/mL) and produced no detectable CPE (Figure 5B,C), consistent with our previous report [28]. Also consistent with those previous findings, complementation of the *cpr1954* null mutant to restore *cpr1954* expression significantly increased sporulation and CPE production back to approximately wild-type levels. Importantly, this study showed for the first time that, when cultured in MDS, the CPR1954KO-1953COM strain overexpressing *cpe1953* in the absence of *cpr1954* makes significantly more spores and CPE than CPR1954KO, producing spores and CPE at near wild-type SM101 levels (Figure 5B,C). Confirming the importance of CPR1953 kinase activity for CPR1954KO-1953COM-induced CPE production, transformation of CPR1953KO with pJIR750-*cpr1953COM*-H430A did not increase sporulation or CPE production (Figure 5B,C) compared to MDS cultures of CPR1954KO.

### 2.4. Overexpression of cpr1953 in the Absence of cpr1954 Expression or Vice Versa, Is Also Sufficient to Obtain near Wild-Type Levels of Sporulation and CPE Production in MIC

Our previous study [28] reported that the contributions of individual OHKs to SM101 sporulation and CPE production can vary between MDS and MIC cultures. Therefore, the vegetative cell viability, sporulation, and CPE production characteristics of the overexpressing strains were also evaluated in overnight MIC cultures. 

**Figure 6:**
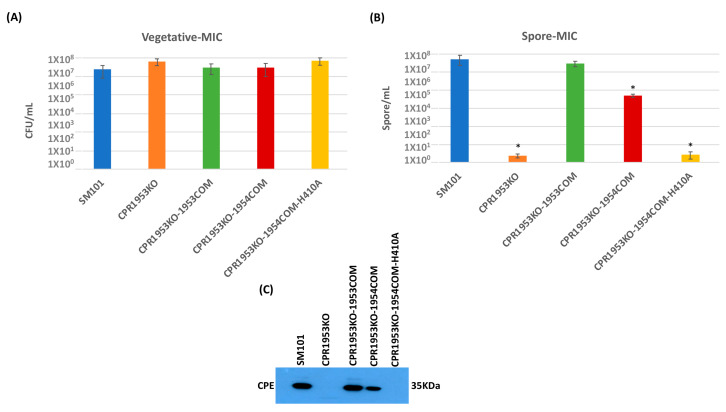
Evaluating the effect of overexpressing the *cpr1953* gene in the absence of *cpr1954* expression of vegetative cell viability, spore formation, and CPE production when SM101 and its derivatives were cultured in MIC. (**A**) Viable vegetative cells (CFU/mL) numbers for SM101, CPR1953KO, CPR1953KO-1953COM, CPR1953KO-1954COM, and CPR1953KO-1954COM-H1410A anaerobically cultured overnight at 37 °C in MIC containing 5% Oxyrase. (**B**) Heat-resistant spore counts (CFU/mL) in aliquots of those same MIC cultures after heating at 70 °C for 20 min to kill the remaining vegetative cells and spore germination induction. (**C**) Western blot analysis of CPE levels in supernatants of those overnight MIC cultures for SM101, CPR1953KO, CPR1953KO-1953COM, CPR1953KO-1954COM, and CPR1953KO-1954COM-H1410A. The size of the protein band is shown at right. Error bars depict SDs. All data were collected from three independent repetition. Asterisk represents *p* < 0.05 versus SM10. Western blot is representative of three independent experiments.

**Figure 7:**
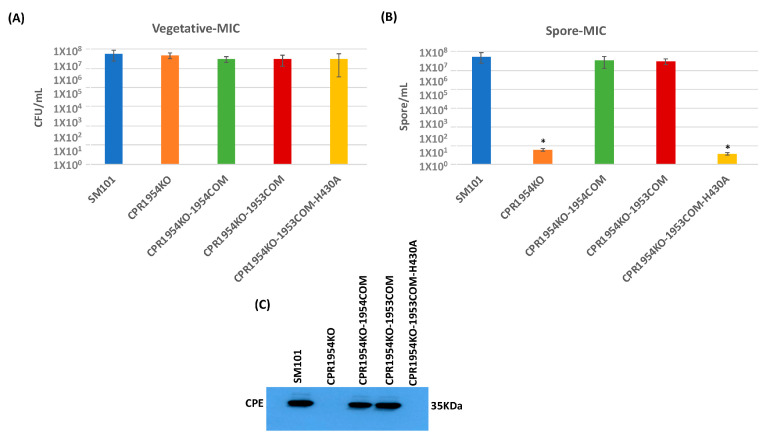
Evaluating the effects of overexpressing the *cpr1954* gene in the absence of *cpr1953* expression on vegetative cell viability, spore formation, and CPE production when SM101 and its derivative isogenic mutant or complementing strains were cultured in MIC. (**A**) Viable vegetative cell numbers (CFU/mL) for SM101, CPR1954KO, CPR1954KO-1954COM, CPR1954KO-1953COM, and CPR1954KO-1953COM-H430A anaerobically cultured overnight at 37 °C in MIC containing 5% Oxyrase. (**B**) Heat-resistant spore counts (CFU/mL) in aliquots of those same MDS cultures after heating at 70 °C for 20 min to kill the remaining vegetative cells and spore germination induction. (**C**) Western blot analysis of CPE levels in supernatants of those overnight MDS cultures from SM101, CPR1954KO, CPR1954KO-1954COM, CPR1954KO-1953COM, and CPR1954KO-1953COM-H430A. The size of the protein band is shown at right. Error bars depict SDs. All data were collected from three independent repetition. Asterisk represents *p* < 0.05 versus SM101. Western blot is representative of three independent experiments.

The results of those analyses indicated that overnight MIC cultures of all isogenic mutants, complementing strains and overexpressing strains contained the same level of viable vegetative cells as similar cultures of the SM101 parental strain (Figure 6A and Figure 7A). As expected from previous results [28], the CPR1953KO and CPR1954KO strains sporulated extremely poorly and did not produce CPE in MIC (Figure 6B,C and Figure 7B,C). However, MIC cultures of the CPR1953-1953COM and CPR1954-1954COM strains showed sporulation and CPE production at near wild-type levels. Importantly, this study showed for the first time that the overexpressing CPR1953KO-1954COM and CPR1954KO-1953COM strains also exhibit near wild-type levels of sporulation and CPE production in overnight MIC culture (Figure 6B,C and Figure 7B,C). In contrast, this study also determined that the CPR1953KO-1954COM-H410 and CPR1954KO-1953COM-H430 control strains did not sporulate or produce CPE (Figure 6B,C and Figure 7B,C).

### 2.5. Analysis of Spo0A Production Levels by Strains Overexpressing cpr1953 in the Absence of cpr1954 Expression or Vice Versa 

To evaluate whether, relative to CPR1953KO or CPR1954KO, the increased sporulation and CPE production observed for CPR1953KO-1954COM and CPR1954KO-1953COM strains might involve increased Spo0A production, wild-type SM101 and its derivatives were grown in MDS broth medium for 3 h at 37 °C. Subsequently, equal amounts of total protein extracted from equal number of cells were subjected to Western blot assay for comparison of strain Spo0A production levels. 

**Figure 8:**
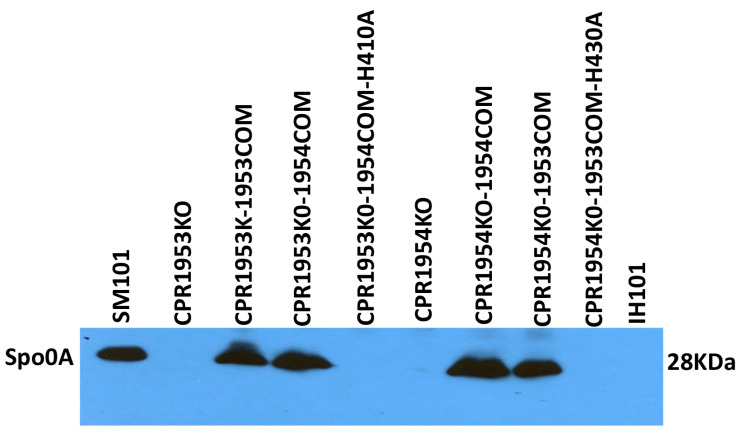
Evaluation the effects of overexpressing *cpr1953* in the absence of *cpr1954* expression or vice versa on Spo0A production in MDS culture. Western blot result for Spo0A production in pelleted cells from 3 h MDS broth medium cultures of wild-type SM101 or its derivatives. The assay was conducted three times, and the result shown is representative of three independent repetitions. The size of protein is shown at right.

Results of this experiment determined that, as reported previously, the SM101 parental strain produces readily detectable levels of Spo0A, while the CPR1953KO and CPR1954KO mutants produce greatly reduced amounts of Spo0A. Furthermore, as also expected from previous results [28], the CPR1953KO-1953COM and CPR1954KO-1954COM complementing strains exhibited near wild-type levels of Spo0A production. Importantly, results shown in Figure 8 also demonstrate for the first time that the overexpressing CPR1953KO-1954COM and CPR1954KO-1953COM strains produce Spo0A at wild-type levels (Figure 8). This effect required the kinase activity of CPR1954 or CPR1953, respectively, since the CPR1953KO-1954COM-H410 and CPR1954KO-1953COM-H430 strains did not produce Spo0A protein. In this study, *spo0A* null mutant (1H101) of SM101 was used as a negative control.

## 3. Discussion

As briefly mentioned in the Introduction, most Bacilli and Clostridia can sporulate in the presence of environmental or metabolic stress signals such as nutrient limitations or population density [18,29,30,31]. In those endospore-forming bacteria, Spo0A phosphorylation initiates sporulation. However, different endospore-formers accomplish Spo0A phosphorylation by varying mechanisms. *Bacillus subtilis*, the paradigm model for sporulation by Bacilli, uses a phosphorelay involving five histidine kinases named KinA-E [23,32,33], with KinA and KinB usually considered as the major kinases for *B. subtilis* sporulation [32,34,35]. KinA and KinB transfer a phosphoryl group to the cognate regulator Spo0F, which is followed by sequential transfer of that phosphoryl group to the phosphotransferase Spo0B and then on to Spo0A [23,32,33,36].

When Clostridial genome sequences first became available, it was believed that all Clostridia lack the classical sporulation phosphorelay of Bacilli. However, the phosphorelay of Bacilli was later identified in some Clostridia [37], although it still has not been found in industrially important, solvent-producing Clostridia or pathogenic Clostridia, including *C. perfringens*. Instead, it was proposed that those Clostridia lacking the classical phosphorelay instead use OHKs to phosphorylate Spo0A and begin their sporulation. As discussed below, support for the OHK/sporulation hypothesis has been obtained for several nonpathogenic solvent-producing Clostridia, where mutants unable to produce particular OHKs showed decreased sporulation [26,28,38,39,40].

Among the pathogenic Clostridia, perhaps the strongest support for the OHK/sporulation hypothesis has been provided by studies of *C. perfringens*. As mentioned in the Introduction, at least five OHKs have been identified that contribute to *C. perfringens* sporulation in MDS, although only two of those OHKs (CPR1953 and CPR1954) also contribute to sporulation in MIC [26,28]. In either MDS or MIC cultures, those two OHKs also had by far the greatest impact on SM101 sporulation of all *C. perfringens* OHKs. Specifically, in either MDS or MIC, CPR1953KO and CPR1954KO producing < 10 spores/mL, which represents a >10^5^-fold reduction in sporulation compared to wild-type SM101 [28]. 

There are some insights into how CPR1953 and CPR1954 affect sporulation and sporulation-dependent CPE production by SM101. CPR1954KO and CPR1953KO were shown to produce substantially reduced levels of Spo0A in 3 h MDS cultures and decreased levels of *spo0A* expression in 3 h MIC culture. Those observations may reflect feedback enhancement of *spo0A* expression by phosphorylated Spo0A [28,41]. A *C. perfringens* OHK named CPR0195 has been shown to directly phosphorylate Spo0A in vitro [26], but this ability has not yet been assessed for CPR1953 or CPR1954. However, Phos-Tag gels did not detect any phosphorylation of the reduced amount of Spo0A produced in 3 h MDS cultures of CPR1954KO, which would be consistent with CPR1954 being required for Spo0A phosphorylation [28]; whether this is a direct or indirect effect of CPR1954 on Spo0A phosphorylation is unclear. Nonetheless, reduced Spo0A production and phosphorylation in the absence of CPR1953 and CPR1954 likely help to explain the profound effects of these OHKs on sporulation. 

Several possible mechanisms could explain why CPR1953 and CPR1954 are both needed, at natural production levels, for SM101 sporulation. For example, these two proteins might represent a novel phosphorelay, where they function together in a step-wise manner. Alternatively, at natural production levels, CPR1953 and CPR1954 might function independently but both be necessary to obtain sufficient levels of Spo0A production and phosphorylation to initiate sporulation. 

To start distinguishing between those two possibilities, the current study examined the effects on sporulation and CPE production when *cpr1954* is overexpressed in a background free of *cpr1953* and vice versa. To accomplish this, we introduced a plasmid carrying the *cpr1954* gene into CPR1953KO, which still carries the wild-type *cpr1954* gene. RT-PCR analyses showed that the resultant CPR1953KO-1954COM strain expressed significantly higher levels of the *cpr1954* gene than CPR1953KO or CPR1953KO-1953COM, consistent with a gene dosage effect increasing *cpr1954* expression in CPR1953KO-1954COM (Figure 3C,D). Similarly, the CPR1954KO-1953COM strain overexpressed *cpr1953* relative to either CPR1954KO or CPR1954KO-1954COM, also consistent with a gene dosage effect increasing c*pr1953* expression in CPR1954KO-1953COM (Figure 3A,B). Characterization of those strains showed that overexpression of either OHK gene, in the absence of expression of the other OHK gene, is sufficient to obtain near wild-type levels of sporulation and CPE production, which is sporulation-dependent. These findings are consistent with a model where CPR1953 and CPR1954 work independently but additively. This model suggests that, at natural production levels an unidentified trigger increases the total kinase activity of both of these OHKs, which leads to sufficient levels of Spo0A production and phosphorylation for SM101 to start sporulation and CPE production. 

The involvement of two major kinases in initiating *C. perfringens* sporulation is somewhat reminiscent of the dual contributions of KinA and KinB to sporulation initiation in *B. subtilis*. However, the strong sporulation impairment exhibited by both the CPR1953KO and CPR1954KO mutants of SM101 contrasts with the partial redundancy of KinA and KinB in *B. subtilis* sporulation, i.e., single *kinA* or *kinB* null mutants show only slight sporulation impairments but a double *kinA/kinB* null mutant exhibits a substantial sporulation deficiency [35]. The situation in some nonpathogenic, solvent-producing Clostridia seems to more closely resemble the apparently independent roles of CPR1953 and CR1954 in regulating *C. perfringens* sporulation. For example, several OHKs increase sporulation in the solvent-producing species *C. acetobutylicum* and those OHKs have been shown to directly phosphorylate Spo0A in vitro [37,39,42]. As this study suggests for CPR1953 and CPR1954 of *C. perfringens*, it appears those *C. acetobutylicum* OHKs function independently, but collectively, to initiate sporulation. Interestingly, those *C. acetobutylicum* OHKs do not appear to be close homologs of CPR1953 and CPR1954, supporting diversity in sporulation initiation amongst the Clostridia. Ongoing further exploration of the mechanistic contributions of CPR1953 and CPR1954 to *C. perfringens* sporulation and CPE production are needed to further elucidate their critical role in these processes. 

## 4. Materials and Methods

### 4.1. C. perfringens Isolates, Plasmids, Media, and Culture Condition 

All *C. perfringens* isolates and plasmids used in this study are listed in Table 1. *C. perfringens* type F strain SM101 was used for this study as the wild-type parental strain. The wild-type strain and all complemented isolates were stored at −20 °C in cooked meat medium (CMM; Oxoid, Hampshire, UK). Stock cultures of all derivatives were made in 35% glycerol and stored at −80 °C. The *C. perfringens* strains were grown anaerobically using a BD GasPak EZ container system at 37 °C in the following broth media: Fluid thioglycollate medium (FTG; Difco Laboratories, Detroit, MI, USA), and Modified Duncan–Strong medium (MDS; 0.4% yeast extract (Becton, Dickinson, Franklin Lakes, NJ, USA), 1.5% proteose peptone [Becton, Dickinson], 1% disodium phosphate heptahydrate (Fisher Scientific, Rockingham County, NH, USA), 0.4% raffinose (Sigma-Aldrich, St. Louis, MO, USA), 0.1% sodium thioglycollate (Sigma-Aldrich), and 1mM caffeine (Sigma-Aldrich)). Brain Heart Infusion (BHI) agar (Research Products International, Mt. Prospect, IL, USA) was used for growth of *C. perfringens* strains on solid media. When required, the culture media were supplemented with chloramphenicol (15 μg/mL) (Sigma-Aldrich) for selection of complementing strains as well as counting of vegetative or spore cells of complementing strains. 

### 4.2. Construction of Overexpressing Strains

To construct the overexpressing strains designated as CPR1953KO-1954COM and CPR1954KO-1953COM, the pJIR750-*cpr1954COM* and pJIR750-*cpr1953COM* plasmids were transferred by electroporation into CPR1953KO or CPR1954KO, respectively, and chloramphenicol resistant (CM^r^) clones were selected. The resultant transformants were confirmed by PCR using the primers listed in Table 2. 

In addition, CPR1953KO-1954COM-H410A and CPR1954KO-1953COM-H430A were constructed by electroporation of pJIR750-*cpr1954COM*-H410A and pJIR750-*cpr1953COM*-H430A plasmids into CPR1953KO or CPR1954KO, respectively. CM^r^ transformants were selected and then confirmed by PCR using the primers listed in Table 2. The pJIR750-*cpr1954COM*-H410A and pJIR750-*cpr1953COM*-H430A plasmids were constructed in our previous study [28]. Briefly, these plasmids encode an alanine substitution for the key functional His residue involved in phosphotransfer for the CPR1953 and CPR1954 orphan histidine kinases. 

### 4.3. Preparation of Pathophysiologically-Relevant Ex Vivo MIC

The ex vivo (mouse intestinal content (MIC)) model used in this study for growth of *C. perfringens* strains was developed previously [28]. Briefly, the small intestinal contents of five healthy male or female BALB/c mice were pooled and then mixed (1:1) with PBS (phosphate buffer saline, Corning, Corning, NY, USA). The diluted sample was then centrifuged at 15,000× *g* for 10 min to eliminate the solid materials. Subsequently, the supernatant was diluted (1:5) with PBS and sterilized by passage through a 0.2 μm syringe filter.

### 4.4. Comparison of Growth Kinetics between C. perfringens Strains Cultured in MDS Sporulation Medium

For comparison of strain growth characteristics in MDS broth, a 200 μL aliquot of an overnight FTG culture of SM101 or its derivative strains was inoculated into 10 mL of freshly prepared MDS medium. The cultures were incubated anaerobically at 37 °C for 0, 2, 4, 6, 8, or 24 h. Then, a 1 mL aliquot culture of each isolate was collected and OD_600_ was measured using a Bio-Rad SmartSpec spectrophotometer (Hercules, CA, USA). This experiment was conducted with three biological replicates.

### 4.5. Measurement of Viable Vegetative Cells or Heat-Resistant Spores 

Quantitative counts of viable vegetative cells or heat-resistant spores of SM101 and its derivative strains were evaluated as previously described [28]. Briefly, for enumeration of viable vegetative cells, a 200 μL or 40 μL aliquot of an overnight FTG of each isolate was added into 10 mL of freshly prepared MDS medium or 1 mL of MIC containing 5% Oxyrase (Oxyrase, Inc., Mansfield, OH, USA), respectively. After overnight anaerobic incubation at 37 °C, those cultures were serially diluted with sterile PBS and plated onto BHI agar plates. The colonies on each BHI agar plates were enumerated after incubation overnight at 37 °C.

To enumerate spore formation, samples were prepared in MDS or MIC as described above. However, before plating the cell suspensions, each culture was heated at 70 °C for 20 min to kill the remaining vegetative cells and induce spore germination. Those heat-shocked samples were then serially diluted with sterile PBS and plated on BHI agar plates. After overnight anaerobic incubation at 37 °C, the colonies were counted. This experiment was conducted with three biological replicates.

### 4.6. Western Blot Analyses of CPE Production

For CPE Western blot analyses, *C. perfringens* cultures were prepared as described earlier in the “measurement of vegetative cells” section. After overnight anaerobic incubation at 37 °C, each MDS or MIC culture was adjusted to equal OD_600_. After this step, those adjusted samples were centrifuged at 15,000× *g* for 5 min. Equal volumes of each supernatant were mixed with 5× concentrated loading buffer containing bromophenol blue dye and SDS (sodium dodecyl sulfate) and then electrophoresed onto a 12% SDS-PAGE gel, followed by transfer onto a polyvinylidene difluoride (PVDF) membrane (Bio-Rad). Subsequently, the blot was blocked at room temperature for 1 h with TBST buffer (Tris buffered saline, 0.1% Tween 20) containing 5% *w*/*v* nonfat dry milk. The blocked blot was then incubated with rabbit polyclonal CPE antibody [44], 1:5000 dilution in TBST buffer containing 5% nonfat dry milk at 4 °C overnight. After 3 washes with TBST buffer for 10 min each, the membrane was incubated with a horseradish peroxidase-conjugated secondary anti-rabbit antibody (Sigma-Aldrich) at dilution of 1:10,000 for 1 h at room temperature. Excess secondary antibody was eliminated by washing 3 times with TBST buffer. Finally, the membrane was developed using SuperSignal West Pico substrate (ThermoFisher, Waltham, MA, USA).

### 4.7. Western Blot Analysis of Spo0A Production

Spo0A Western blot analysis was performed as previously described [28]. Briefly, wild-type SM101 and its derivative strains were grown in 10 mL of freshly prepared MDS medium for 3 h at 37 °C. Each *C. perfringens* culture was then adjusted to equal OD_600_ using a Bio-Rad SmartSpec spectrophotometer. Equal volume of those adjusted cultures was harvested by centrifugation, washed with PBS buffer, lysed with B-PER reagent (ThermoFisher Scientific) containing protease inhibitor cocktail II (Research Products International), and then boiled for 5 min at 95 °C. The whole-cell extracts were then subjected to 12% SDS-PAGE followed by Western blot with rabbit polyclonal antibody made against *Clostridioides difficile* Spo0A at dilution of 1:2000. The primary antibody was detected by horseradish peroxidase-conjugated secondary anti-rabbit antibody at dilution of 1:10,000, and Western blot was visualized using SuperSignal West Pico substrate. In this experiment, the *spo0A* knock-out mutant 1H101 [22] was served as a negative control.

### 4.8. DNA Extraction and PCR Analysis 

Genomic DNA was extracted from wild-type SM101 and its derivative strains using the MasterPure Gram-positive bacterial DNA purification kit (Epicentre, Paris, France). All previously constructed [28] plasmids used in this study were isolated from *C. perfringens* type A strain 13, the cloning host strain, using a QIAprep Spin Miniprep Kit (Qiagen, Hilden, Germany), according to the manufacturer’s instructions. 

The PCR reactions for confirming the isogenic mutants and complemented strains were conducted using 2× Dream*Taq* Green PCR Master Mix (ThermoFisher Scientific) and appropriate primers listed in Table 2. The following PCR amplification condition was used for this experiment: (1) 95 °C for 5 min; (2) 35 cycles of 95 °C for 30 s, 55 °C for 30 s, 72 °C for 90 s, and (3) a final extension for 5 min at 72 °C.

In addition, LongAmp *Taq* polymerase (New England Biolabs, Ipswich, MA, USA) was used for confirmation of CPR1953KO-1954COM, CPR1954KO-1953COM, CPR1953KO-1954COM-H410A, and CPR1954KO-1953COM-H430A constructs. The following PCR parameters were used: (1) 95 °C for 5 min; (2) 35 cycles of 95 °C for 30 s, 55 °C for 30 s, 65 °C for 50 s per Kb, and (3) a final extension for 10 min at 65 °C. Finally, an aliquot of each PCR reaction (20 μL) was electrophoresed on a 2% agarose gel and then visualized after staining with ethidium bromide.

### 4.9. RNA Isolation and RT-PCR Analysis 

A 200 μL aliquot of an overnight FTG culture of SM101 or its derivative strains was transferred into 10 mL of MDS broth and anaerobically incubated at 37 °C for 3 h. Total RNA was isolated from pelleted cells using the saturated phenol (Fisher Scientific) protocol, as previously described [45]. Purified RNA was quantified by NanoDrop spectrophotometer (ThermoFisher Scientific) and then stored in a −80 °C freezer. Furthermore, the purity and quality of each extracted RNA sample was evaluated by conducting PCR assay for *polC* and *16S* genes in the absence or presence of reverse transcriptase enzyme. RT-PCR assay was performed using one-step RT-PCR containing 200 ng of purified RNA, avian myeloblastosis virus (AMV) reverse transcriptase (4 U; Promega, Madison, WI, USA), RNA and DNA free dH_2_O, 2× DreamTaq Green PCR Master Mix (ThermoFisher Scientific), and appropriate primer sets listed in Table 2. Finally, the RT-PCR assay was carried out using the following parameters: (1) 95 °C for 4 min; (2) 42 °C for 45 min (for cDNA synthesis); (3) 95 °C for 2 min; (4) 30 cycles of 95 °C for 30 s, 55 °C for 30 s, 72 °C for 30 s, and (5) a final extension for 5 min at 72 °C. 

### 4.10. Statistical Analysis 

Differences among the results were determined by using either ordinary one-way analysis of variance (ANOVA; GraphPad Prism 8) or Student’s unpaired *t* test from at least three independent experiments. If the *p* value was less than 0.05, the result was considered statistically significant.

## Figures and Tables

**Table 1:** Strains and plasmids used in this study.

Strains or Plasmids	Description	Reference
**Strains**		
SM101	Transformable derivative of a type F food poisoning strain	[17]
Strain 13	Soil isolate	[25]
CPR1953KO	SM101 *cpr1953* null mutant	[28]
CPR1954KO	SM101 *cpr1954* null mutant	[28]
CPR1953KO-1953COM	SM101 *cpr1953* null mutant complement with *cpr1953*	[28]
CPR1954KO-1954COM	SM101 *cpr1954* null mutant complemented with *cpr1954*	[28]
CPR1953KO-1954COM	SM101 *cpr1953* null mutant complement with *cpr1954*	This study
CPR1954KO-1953COM	SM101 *cpr1954* null mutant complemented with *cpr1953*	This study
CPR1953KO-1954COM-H410A	SM101 *cpr1953* null mutant complemented with *cpr1954*-H410A	This study
CPR1954KO-1953COM-H430A	SM101 *cpr1954* null mutant complemented with *cpr1953*-H430A	This study
**Plasmids**		
pJIR750	*C. perfringens*–*E. coli* shuttle plasmid	[43]
pJIR750-*cpr1953COM*	Used for construction of CPR1954KO-1953COM	[28]
pJIR750-*cpr1954COM*	Used for construction of CPR1953KO-1954COM	[28]
pJIR750-*cpr1953COM*-H430A	Used for construction of CPR1954KO-1953COM-H430A	[28]
pJIR750-*cpr1954COM*-H410A	Used for construction of CPR1953KO-1954COM-H410A	[28]
pUC57	*E. coli* cloning vector	GenScript
pUC57-*cpr1953COM*-H430A	Used for construction of CPR1953COM-H430A plasmid	[28]
pUC57-*cpr1954COM*-H410A	Used for construction of a CPR1954COM-H410A plasmid	[28]

**Table 2:** Primers used in this study.

Primer Name	Purpose	Sequence (5′ -> 3′)	PCR Product Size (bp)
1953KO-F	Screen for intron insertion in *cpr1953*; *cpr1953* RT-PCR; Screen for CPR1953KO-1953COM; CPR1953KO-1954COM and CPR1953KO-1954COM-H410A	GTGGAATACAAAGTGGAATATGA	Wild-type: (365); Mutant: (1265)
1953KO-R		CCTAATGCTATAACAAATACAATAA	
1954KO-F	Screen for intron insertion in *cpr1954*; *cpr1954* RT-PCR; Screen for CPR1954KO-1954COM; CPR1954KO-1953COM and CPR195KO-1953COM-H430A	GATGTAATTGTGTTTGGGGTGAT	Wild-type: (596); Mutant: (1496)
1954KO-R		CCTGAAGAATATGAAGCTTCTCC	
polC-F	RT-PCR analysis of *polC*	ACTTCCCTGCAAGCCTCTTCTCCT	300
polC-R		TGGTTCAGCTTGTGAAGCAGGGC	
16S-F	RT-PCR analysis of *16S*	CCTTACCTACACTTGACATCCC	118
16S-R		GGACTTAACCCAACATCTCACG	
PJIR750-F	Screen for CPR1954KO-1953COM and CPR1954KO-1953COM-H430A strains	GTTGGCCGATTCATTAATGCA	1354
CPR1953-R		GAATAAGAAGCCAAAATATCCCTA	
PJIR750-F	Screen for CPR1953KO-1954COM and CPR1953KO-1954COM-H410A strains	GTTGGCCGATTCATTAATGCA	2300
CPR1954-R		TCTTTAAGTCATGAGATATGCTTGA	

## Data Availability

All the data that support the findings of this study are available from the corresponding author upon reasonable request.
